# Characterising the immune profile of the kidney biopsy at lupus nephritis flare differentiates early treatment responders from non-responders

**DOI:** 10.1136/lupus-2015-000112

**Published:** 2015-11-18

**Authors:** Samir V Parikh, Ana Malvar, Huijuan Song, Valeria Alberton, Bruno Lococo, Jay Vance, Jianying Zhang, Lianbo Yu, Brad H Rovin

**Affiliations:** 1 Division of Nephrology, The Ohio State University Wexner Medical Center, Columbus, Ohio, USA; 2 Nephrology Unit, Hospital Fernandez, Buenos Aires, Argentina; 3 Department of Pathology, Hospital Fernandez, Buenos Aires, Argentina; 4 Center for Biostatistics, The Ohio State University Wexner Medical Center, Columbus, Ohio, USA

**Keywords:** Lupus Nephritis, Systemic Lupus Erythematosus, Treatment, Autoimmunity

## Abstract

**Introduction:**

The kidney biopsy is used to diagnose and guide initial therapy in patients with lupus nephritis (LN). Kidney histology does not correlate well with clinical measurements of kidney injury or predict how patients will respond to standard-of-care immunosuppression. We postulated that the gene expression profile of kidney tissue at the time of biopsy may differentiate patients who will from those who will not respond to treatment.

**Methods:**

The expression of 511 immune-response genes was measured in kidney biopsies from 19 patients with proliferative LN and 4 normal controls. RNA was extracted from formalin-fixed, paraffin-embedded kidney biopsies done at flare. After induction therapy, 5 patients achieved a complete clinical response (CR), 10 had a partial response (PR) and 4 patients were non-responders (NRs). Transcript expression was compared with normal controls and between renal response groups.

**Results:**

A principal component analysis showed that intrarenal transcript expression from normal kidney, CR biopsies and NR biopsies segregated from each other. The top genes responsible for CR clustering included several interferon pathway genes (*STAT1*, *IRF1*, *IRF7*, *MX1*, *STAT2*, *JAK2*), while complement genes (*C1R*, *C1QB*, *C6*, *C9*, *C5*, *MASP2*) were mainly responsible for NR clustering. Overall, 35 genes were uniquely expressed in NR compared with CR. Pathway analysis revealed that interferon signalling and complement activation pathways were upregulated in both groups, while BAFF, APRIL, nuclear factor-κB and interleukin-6 signalling were increased in CR but suppressed in NR.

**Conclusions:**

These data suggest that molecular profiling of the kidney biopsy at LN flare may be useful in predicting treatment response to induction therapy.

**Key messages**

- Not all LN is created equal: The molecular profile of LN flares that end in complete response differs from flares that end in no response.
- Molecular profiling of the kidney biopsy at LN flare may help guide treatment and better predict response.
- The addition of molecular analysis to routine histology will help facilitate the personalization of LN treatment.

## Introduction

The percutaneous kidney biopsy is the gold standard for the diagnosis of glomerular diseases. Perhaps more so than for any other glomerular disease, biopsy findings are used to classify and subgroup lupus nephritis (LN) in order to better inform treatment decisions and predict prognosis.^1^ ^2^ Several schemas have been used to classify LN biopsies, the most recent being the 2004 International Society of Nephrology (ISN) and Renal Pathology Society (RPS) classification.^2^ ^3^ Although the objective of the ISN/RPS classification was to align histology with outcomes,^3–5^ little progress has been made in using the kidney biopsy to predict treatment response in LN. Contributing to this is the poor correlation between clinical findings and renal histology.^6–9^ A possible explanation for the discordance between clinical and histological findings is that the histological responses of the kidney to injury are limited, whereas the pathogenic mechanisms of renal injury in LN are diverse. It is likely that molecular analysis of kidney biopsies will provide more information about how the kidney will respond to treatment than histology alone.

To test this hypothesis, we measured transcript expression of a panel of immune response genes in the diagnostic kidney biopsies of patients with proliferative LN and evaluated their expression profile at flare. Gene expression profiles were compared between those who achieved a complete clinical response and those who did not achieve a response after standard-of-care LN induction therapy. Differentially expressed genes were subject to informatics analyses to predict the immune signalling pathways that differentiated responders and non-responders (NRs) at the start of treatment.

## Methods

### Kidney biopsies

For this proof-of-concept study, transcript expression was measured in the kidney biopsies of 19 patients with proliferative (class III or IV±V) LN. These biopsies were done from 2007 to 2011. The biopsies had been archived after all clinical testing was completed. The use of these biopsies was approved by the Hospital Fernandez ethics board.

As a control, archived kidney tissue from pre-implantation biopsies of living-donor kidneys (n=4) was analysed in parallel with the LN biopsies. Pre-implantation biopsies are done on all donor kidneys at the Ohio State University Wexner Medical Center as part of the clinical transplant protocol. The use of these biopsies was approved by The Ohio State University Institutional Review Board**.**

### Treatment protocols and outcomes

All patients were treated with standard-of-care immunosuppression protocols. Sixty-three per cent of the cohort received 2000–3000 mg/day mycophenolate mofitel, while 37% were given 750–1000 mg/month of intravenous cyclophosphamide. All patients received a corticosteroid taper starting with 1 mg/kg/day prednisone at the beginning of induction. The induction period lasted, in general, 6 months.

The serum creatinine (SCr) concentration and 24 h urine protein level were available on all patients at flare and after finishing induction treatment. Complete renal response was defined as having an improvement in proteinuria to <0.5 g/day with normalisation of SCr. Partial renal response (PR) was defined as at least a 50% reduction in proteinuria, to a level <3 g/day, but >0.5 g/day, with stable or improved SCr.^10^ Patients who did not meet either of these criteria were defined as NRs.

### RNA extraction and analysis

All biopsies used in this study had been formalin-fixed and paraffin-embedded (FFPE). Ten micron sections were cut from the paraffin blocks, and for each biopsy two sections were deparaffinised and digested with proteinase K. DNA was removed with DNase. RNA was precipitated and the precipitate was added to RNeasy MinElute spin columns (Qiagen, Redwood City, California, USA). RNA was eluted in RNase-free water. The complete details of FFPE deparaffinisation and RNA extraction can be found in online supplementary methods 1.

Gene transcript expression was analysed from 250 ng of extracted RNA using the Nanostring ncounter platform and the GX human immunology transcript panel (Nanostring Technologies, Seattle, Washington, USA).^11–13^ The ncounter platform was chosen because it is superior to microarray for quantification of gene expression in FFPE samples.^14^ ^15^ The human immunology panel consisted of 511 immune response genes, 6 positive control genes and 6 negative control genes. A complete list of these genes can be found in online supplementary table S1.

Multiplex RT-PCR using TaqMan Gene expression assays (Applied Biosystems/Life Technology, Grand Island, New York, USA, catalogue # 4384267) was done to verify Nanostring results for a subset of differentially expressed transcripts. Using a high-capacity RNA-to-cDNA kit (Applied Biosystems/Life Technology, catalogue # 4387406), cDNA was generated from 300 ng total tissue RNA and data were collected during RT-PCR cycles using gene-specific fluorogenic probes. The complete details of the RT-PCR protocol can be found in online supplementary methods 2.

### Statistical analysis

Before statistical analyses, raw gene expression data were normalised to the positive spike-in controls and then log2 transformed. To reduce the false positive rate, only genes with an expression level at least 2 SDs above the mean expression of the negative controls were included in the analysis. Quantile normalisation was used for normalisation across samples. Overall, 382 transcripts survived normalisation and were further analysed for differential expression.

Patients were stratified by response status (CR, PR and NR). Transcript expression levels were compared with normal kidney tissue and between responder groups to identify gene signatures and/or pathways that differentiated responders from NRs.

Descriptive statistics are presented as mean±SD or as a percentage. For clinical variables, t tests, analysis of variance model or Wilcoxon rank-sum tests were applied as appropriate, followed by Bonferroni correction for multiple comparisons. For categorical clinical variables, Fisher's exact test was used. Adjusted p values were reported for each comparison and were considered significant if <0.05.

A linear model was used to compare the gene expression of normal kidney tissue to LN biopsies from patients who achieved a CR, PR or NR after induction therapy, and also to directly compare gene expression between the three LN responder groups. In order to improve the estimates of variability and differential expression, variance smoothing methods were employed.^16^ p Values were adjusted by controlling the mean number of false positives at 4 out of 400 genes (ie, α=0.01). To be considered differentially expressed, at least a twofold difference in transcript levels and a p value <0.01 were required for any specific gene.

The RNA analysis for this cohort was conducted in two batches with 9 samples in the first batch and 10 samples in the second batch. The control samples were the same for both batches. To prevent confounding from batch effect, a batch effect adjustment was applied for testing expression differences in the linear model and to standardise the data across the batches.

### Pathway analysis

Ingenuity Pathway Analysis (IPA, Qiagen) was used to identify canonical pathways that were differentially expressed between the treatment response groups and controls, and between each treatment response group. For each comparison, all 382 genes were analysed for significance. For IPA analysis, transcripts with a p value <0.05 and at least a 1.5-fold change were included in the analysis. These criteria were used to include as many significant differentially expressed and biologically relevant genes as possible to enrich pathway analyses. Comparing CR to control, 68 transcripts met the criteria and were included in the analysis. Comparing NR to control, 138 transcripts met the criteria and were included in the analysis. The Ingenuity Knowledge Base was used as the reference set against which the significant transcripts were compared for enrichment. Significance of upregulated or downregulated pathways was determined using Fisher's exact test and is presented as the negative logarithm of the p value (−log (p value)). A multiple corrections test is not available for IPA; therefore, all values are reported as unadjusted p values. The predicted activation state (upregulated or downregulated) of significantly expressed pathways was determined by a z-score algorithm that compared the gene expression data set with the expected canonical pathway patterns (http://ingenuity.force.com/ipa).

## Results

### Characteristics of the LN cohort

The demographic, clinical and pathological characteristics of the LN patients segregated by renal response after initial therapy are given in table 1. The median time to follow-up was 10 months (range 6–37 months). All patients were Caucasian and Hispanic. Although the clinical and histological findings at biopsy were generally similar between response groups, NR had more proteinuria at flare. Additionally, 40% of the flares in the CR group were relapses, while in NR 75% were relapses (p=NS). For relapsing patients, their prior flares occurred two or more years prior to the current flare event. Immunosuppressive treatment prior to the kidney biopsy was similar between the groups. Two patients in the CR group were on azathioprine prior to the kidney biopsy while three patients were on prednisone only (≤10 mg/day). In the NR group, one patient was not on any immunosuppressive therapy prior to biopsy, two were on prednisone only (≤10 mg/day) and one patient was on azathioprine. For the PR group, four patients were off immunosuppression at the time of kidney biopsy, four patients were on prednisone only (≤20 mg/day) and two patients were on azathioprine. Activity and chronicity indices were not significantly different between the responder groups. After induction treatment, SCr and proteinuria improved in the CR and PR groups, but SCr worsened in the NR group.

**Table 1:** Demographic and clinical parameters of the cohort

	CR (n=5)	PR (n=10)	NR (n=4)	p Value (CR vs NR)	p Value (CR vs PR	p Value (PR vs NR)
Age (years)	24.8±4.97	34±5.35	30±8.16	NS	0.035*	NS
Female (%)	4 (80)	8 (80)	4 (100)	NS	NS	NS
Class IV (%)	3 (60)	9 (90)	4 (100)	NS	NS	NS
No treatment prior to biopsy (%)†	0%	40%	25%	NS	NS	NS
Cyclophosphamide induction (%)	2 (40)	3 (30)	2 (50)	NS	NS	NS
MMF induction (%)	3 (60)	7 (70)	2 (50)	NS	NS	NS
SCr‡ at diagnosis	0.94±0.31	1.08±0.53	1.08±0.28	NS	NS	NS
SCr after induction	0.74±0.15	0.81±0.12	1.18±0.36	0.0129	NS	0.018
Proteinuria§ at diagnosis	3.04±1.11	4.38±1.09	5.5±2.08	0.043	NS	NS
Proteinuria after induction	0.24±0.09	1.49±0.51	3.33±1.27	<0.0001	0.0114	0.0009
First flare (%)	3 (60%)	8 (80%)	1 (25%)	NS	NS	NS
Median activity index (range)	6 (4–8)	8 (4–12)	5 (4–12)	NS	NS	NS
Median chronicity index (range)	2 (0–4)	4 (2–5)	5 (0–6)	NS	NS	NS

*For clinical variables t test, analysis of variance or Wilcoxon rank-sum tests were done, followed by Bonferroni post hoc testing for multiple comparisons as appropriate.

†Patients on treatment for systemic lupus erythematosus were on either azathioprine and/or prednisone (see text).

‡Serum creatinine concentration in mg/dL.

§Urine protein in g/day.

CR, complete renal response group; MMF, mycophenolate mofitel; NS, not significant; NR, non-responder group; PR, partial renal response group.

**Figure 1:**
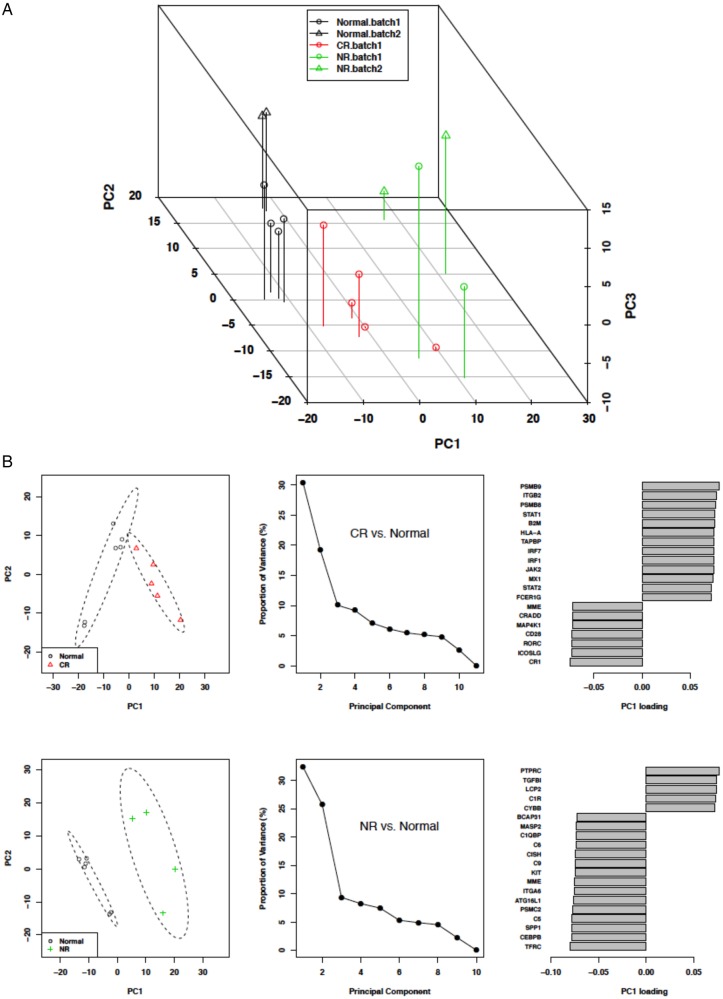
Principal component analysis (PCA) of immune response gene expression in kidney biopsies. (A) The PCA based on gene expression data for complete responders (CRs), non-responders (NRs) and normal controls. The RNA analysis of this biopsy set was conducted in two batches. A batch effect adjustment was applied to prevent confounding and both batches are represented in the figure (batch 1, circles; batch 2, triangles). The normal controls were common to both batches. The PCA shows that CR (except for one patient), NR and normal controls groups clustered separately from each other. (B) A factor loading plot using flare data from CR and NR to identify the genes important for the clustering seen in the PCA. Principal component 1 (PC1) was used as it accounted for the highest proportion (35–50%) of variance. For each PC1 loading plot, only the top 20 genes ranked by absolute factor loadings for PC1 were selected. The plots show the top genes contributing to group clustering.

A principal component analysis (PCA) of immune gene transcripts from normal kidney and the LN responder groups was done (figure 1A). This showed that normal controls clustered together and were separated from the LN responder groups. Additionally, all but one CR patient clustered together and separately from NR. The individual patients of the PR group did not cluster, but instead were distributed between the CR and NR clusters (data not shown).

Factor-loading plots for each principal component were created to identify the genes contributing to group clustering.^17^ Because principal component 1 (PC1) seemed to best associate with group clustering, the top 20 genes for PC1 were determined after batch adjustment (figure 1B). The interferon-inducible genes *STAT1*, *IRF1*, *IRF7*, *MX1*, *STAT2* and *JAK2* contributed prominently to CR clustering. In addition, the proteasome genes *PSMB8* and *PSMB9*, known to be induced by gamma interferon,^18^ and the T cell co-stimulation genes *CD28* and *ICOSLG* were important for CR clustering.

Contributing significantly to NR clustering were genes for the complement components *C1QBP*, *C1R*, *MASP*, *C6*, *C9* and *C5.* Additionally, *TGFBI, CEBPB* and *SPP1* were also important for NR clustering (figure 1B)*. TGFBI* is known to promote renal fibrosis and has previously been implicated in LN.^19–21^ *CEBPB* encodes the CCAAT/enhancer binding protein-β and is necessary for macrophage-mediated removal of apoptotic debris.^22^ Osteopontin (*SPP1*) is produced by various immune cells and is important for regulating several aspects of the immune system including T-helper cell balance and B cell production of antibodies.^23^ Overexpression of osteopontin has been implicated in the development of murine LN^24^ and is associated with human systemic lupus erythematosus (SLE).^25^

### Immune gene expression profiles in kidney biopsies at LN flare

**Figure 2:**
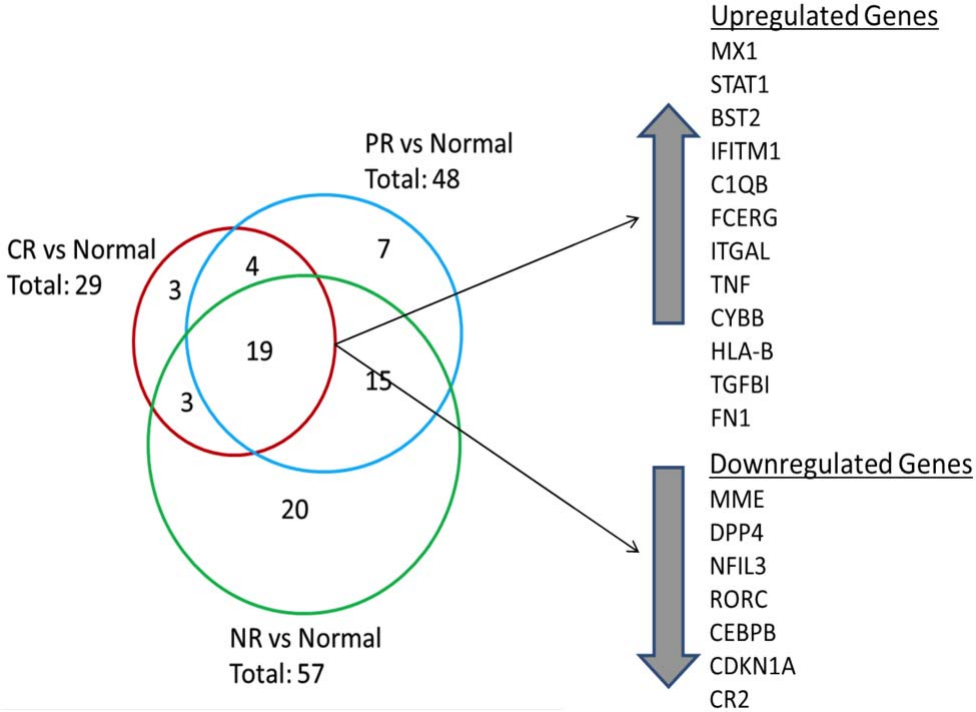
Differential renal gene expression at flare for each lupus nephritis (LN) responder group compared with normal kidney. The Venn diagram shows the number of common and unique genes in each LN responder group. The 19 genes that were differentially expressed between all LN groups and normal tissue are listed. Twelve genes were upregulated and seven genes were downregulated. Three genes were uniquely expressed in the CR group compared with normal while seven genes were uniquely expressed in the partial response (PR) group. A 20-gene signature differentiated non-responders (NR) from CR and PR groups.

Overall, 71 transcripts were differentially expressed in LN kidneys compared with normal control kidneys (figure 2). Of these, 19 transcripts were common to each LN response type (figure 2). Transcripts with altered expression in LN that were unique to each response group are listed in table 2.

**Table 2:** Differentially expressed transcripts at flare in each lupus nephritis (LN) response group compared with normal controls

Complete response group			No response group			Partial response group		
Gene	Fold change*	p Value†	Gene	Fold change*	p Value†	Gene	Fold change*	p Value†
*HLA-A*	2.17	0.0007	*LILRA3*	2.23	0.0006	*CFB*	2.2	0
*IFIH1*	2.16	0.0003	*CSF2RB*	2.23	0	*IL18R1*	0.46	0
*NFATC1*	0.48	0.0002	*TAGAP*	2.21	0.0004	*KIR2DL5A*	0.46	0.0108
			*BTK*	2.17	0.0004	*NFKBIA*	0.44	0
			*IL1RN*	2.11	0.0099	*TAL1*	0.41	0
			*CX3CR1*	2.07	0.0022	*IL1RL1*	0.41	0.0011
			*LCP2*	2.01	0.0001	*FKBP5*	0.11	0
			*FCGR2C*	2.01	0.0005			
			*IGF2R*	0.49	0			
			*NOS2*	0.49	0.0013			
			*RELB*	0.49	0.0118			
			*FADD*	0.47	0			
			*PLAU4*	0.47	0.0029			
			*ITGA6*	0.46	0			
			*CD274*	0.46	0.0001			
			*IL6R*	0.46	0.0001			
			*IL6ST*	0.46	0.0029			
			*ICOSLG*	0.45	0			
			*BST1*	0.44	0.0003			
			*CD81*	0.41	0			

*LN compared with normal.

†LN compared with normal.

CR renal tissue showed three unique transcripts, including *human leukocyte antigen* (*HLA-A*) and *interferon-induced helicase C domain protein 1* (*IFIH1*), which were upregulated relative to control, and *nuclear factor of activated T cells*, *cytoplasmic 1* (*NFATC1*), which was downregulated. *IFIH1* is a cytoplasmic dsRNA sensor important for activating interferon-alpha, and has been shown to promote apoptosis, inflammation and autoantibody production in SLE.^26^ *NFATC1* regulates T cell proliferation and differentiation and can be blocked by calcineurin inhibitors.^27^

Seven transcripts were unique to PR, including increased expression of *complement factor B* (*CFB*), and decreased expression of *interleukin-1 receptor-like-1* (*IL1RL1*) and *FK506 binding protein* (*FKBP5*) (table 2). *IL1RL1* is an IL-1 family member that binds IL-33 and regulates nuclear factor (NF)-κB-mediated Th2 immune responses.^28^ *FKBP5* is an immunophilin family member that binds tacrolimus and rapamycin and is important for immune regulation.

Twenty transcripts were unique to NR, of which 12 were decreased relative to control and 8 were increased (table 2). Transcripts with decreased expression included *fas-associated protein with death domain* (*FADD*), a regulator of apoptosis, *programmed-death ligand 1* (*CD274/PD-L1*), which regulates autoreactive T cell production,^29^ *interleukin-6 signal transducer* (*IL-6ST*) and *IL-6 receptor* (*IL-6R*). Transcripts with increased expression in NR kidneys included *IL-1 receptor antagonist* (*IL1RN*), a natural antagonist of IL-1, chemokine *C-X3-C motif receptor 1* (*CXCR1*), the receptor for a chemokine involved in migration and adhesion of leucocytes, and *T cell activation GTPase activating protein* (*TAGAP*), which plays a role in T cell activation.^30^

When CR and NR were directly compared, five transcripts were found to be differentially expressed between these two extremes of clinical response. *Membrane metalloendopeptidase* (*MME*) (p=0.0034), a glycoprotein abundant in the proximal tubule of the kidney, *FADD* (p<0.0001) and *CD274/PD-L1* (p=0.0002) were ≥2-fold higher in CR. Complement component *C1S* (p=0.003) and *integrin beta-2* (ITGB2) (p=0.0007) were ≥2-fold lower in CR versus NR.

### RT-PCR measurement of selected transcripts

*MME*, *ITGB2*, *MX1*, *STAT1* and *CCL19* mRNA levels were measured by RT-PCR and compared with the results obtained using the ncounter platform on the same biopsies to confirm trends identified by Nanostring. The selected transcripts were found to be differentially expressed between LN groups and controls by Nanostring, and the same trend in transcript expression was observed using RT-PCR (see online supplementary table S2).

### Differentially regulated pathways at flare

**Figure 3:**
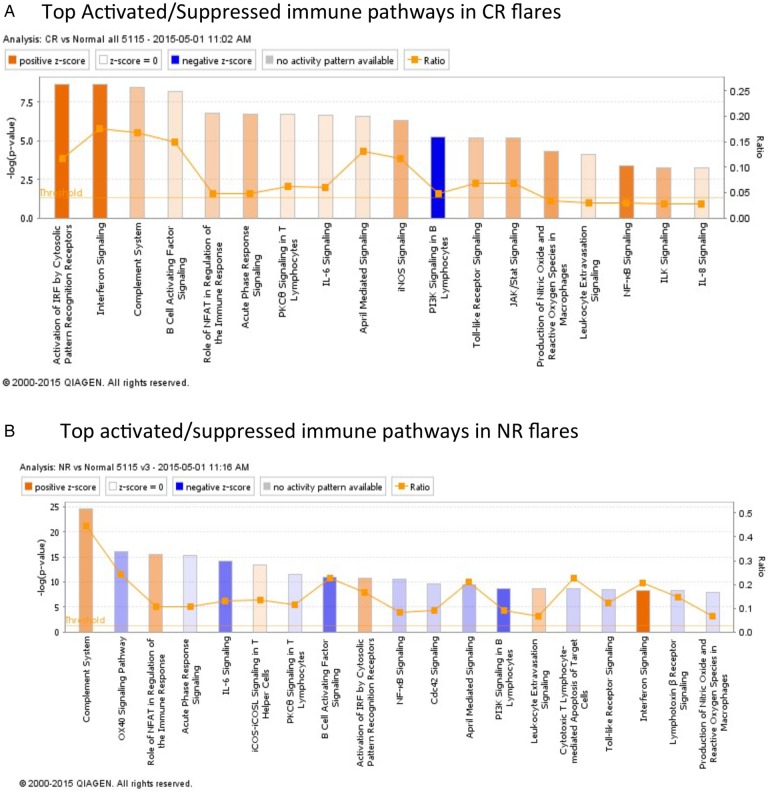
Canonical immune pathway expression in complete responders (CR) and non-responders (NRs) compared with normal controls at flare. Using Ingenuity Pathway Analysis (IPA), the lupus nephritis (LN) flare groups were compared with normal kidney based on differentially expressed genes. Only canonical pathways where activation status could be determined by IPA were included. The bars reflect the p value for each pathway. The p value measures the likelihood that association between the differentially expressed genes in the data set and the pathway is due to random chance. The smaller the p value, the taller the bar in the figure, and the less likely the association is due to random chance. All the pathways represented had p values <0.05 by right-tailed Fisher's exact test and are considered statistically significant. (A) Pathways that were predicted to be activated or suppressed in CR flares compared with normal controls. (B) Pathways that were predicted to be activated or suppressed in NR flares compared with normal controls.

To integrate all of the differentially expressed tissue transcripts into immunological pathways that may be relevant to kidney injury in LN, the expression differences of the genes listed in table 2 were analysed by IPA and differentially activated canonical signalling pathways were identified. Figure 3 shows the top differentially activated or suppressed pathways in CR and NR compared with control. Only immune pathways where an activation status could be predicted were reported. Interferon signalling, complement and leucocyte extravasation pathways were predicted to be activated in CR and NR, while PI3K signalling in B-lymphocytes was predicted to be suppressed (figure 3). Pathway analysis also identified several differences between CR and NR. For example, in CR kidneys IL-6, NF-κB, B cell activating factor and toll-like receptor (TLR) signalling were predicted to be activated, whereas in NR kidneys these same pathways were predicted to be suppressed (figure 3).

## Discussion

The present work shows that the intrarenal transcript expression profile of the diagnostic kidney biopsy in LN differs between patients who had a rapid clinical response to induction therapy and patients who did not. This is the first study to examine the association of future clinical renal response with the molecular profile of the human LN kidney.

A subset of 19 immune response genes from the transcript panel used in this study was differentially expressed in all LN biopsies compared with normal kidney tissue. The protein products of these genes are consistent with our current understanding of how the immune system is dysregulated in SLE. For example, the expression of interferon and complement genes was significantly increased in LN kidneys, and both pathways appear to be involved in the pathogenesis of SLE and LN.^31–38^ Conversely, the expression of *CEBPB* was decreased in LN kidneys. Because macrophages lacking *CEBPB* do not remove apoptotic debris very well, such debris may accumulate and contribute to kidney-specific autoimmunity and autoantibody production.^22^ ^39–41^

PCA of transcript expression revealed clustering of LN flare groups compared with controls. Factor loading plots of the PCA identified genes that were responsible for the separation in both CR and NR. Interferon-inducible genes appeared to be most responsible for CR clustering while complement genes seemed most responsible for clustering in NR.

Direct comparison between CR and NR transcript expression at flare yielded five differentially expressed genes. The composition of this panel suggests that the function or activity of several immune pathways may distinguish CR from NR. *FADD* was suppressed in NR compared with CR. *FADD* binds Fas receptor after it is engaged by *fas*-ligand (FasL) and activates an apoptosis cascade through procaspases 8 and 10. Fas/FasL has previously been shown to be important for maintaining immune tolerance through elimination of autoreactive lymphocytes.^42^ In murine models, Fas/FasL eradicate autoreactive T and B cells from germinal centres of secondary lymphoid organs and deficiency in Fas or FasL leads to lupus-like disease.^43^ ^44^ Decreased *FADD* expression in NR may contribute to blunted tolerance and inefficient clearing of apoptotic debris compared with CR flares.

CD274/PD-L1 is a transmembrane protein that interacts with the PD-1 receptor and regulates T cell co-stimulation. It was suppressed in NR relative to CR. The PD-1:PD-L1 pathway is known to eliminate autoreactive T cells and protect against autoimmunity.^45^ PD-1^−/−^ mice develop lupus-like disease including glomerulonephritis.^46^ Additionally, *CD274/PD-L1* has been shown to play an important role in T regulatory cell generation from naive CD4 T cells.^47^ In human SLE, *CD274/PD-L1* is suppressed in flare but returns when disease is in remission.^29^ This suggests that suppression of *CD274/PD-L1* as seen in NR may lead to unregulated autoreactive T cell activation and defective tolerance compared with CR.

Complement is involved in all LN flares; however, the increased expression of C1 components suggests the classical pathway may be more active in NR flares. Finally, increased expression of *ITGB2*, the integrin beta-chain subunit for LFA-1, in NR compared with CR suggests that DNA hypomethylation and unregulated T cell activity may be more prominent in LN flares that end in NR.^48^ LFA-1 overexpression is associated with T cell DNA hypomethylation and has previously been implicated in promoting autoreactivity and lupus-like disease in experimental animals.^49^

In a second approach to distinguish future CR from NR using the initial diagnostic biopsy, transcript profiles from each group were analysed to identify differentially activated pathways of immune-mediated kidney injury. CR biopsies at flare were characterised by activation of pro-inflammatory (IL-6, NF-κB, TLR signalling) and B cell (BAFF and APRIL) pathways. These data are consistent with the presumed pathogenesis of SLE and LN. In contrast, and unexpectedly, while interferon and complement were upregulated in NR, the same pro-inflammatory and B cell pathways activated in CR were suppressed in NR kidneys.

There are several potential reasons for the molecular differences between CR and NR kidneys at flare. While immunosuppressive medications at the time of biopsy could be confounders, the NR and CR patients were on similar types and levels of therapy at flare, so this seems unlikely. The simplest explanation is that more CR patients were new onset LN compared with NR, which was mainly relapsing LN. Although not statistically significant, NR had more chronic kidney damage than CR, as reflected by the higher average value of the chronicity index. While it is not clear how chronic parenchymal injury may modify the acute inflammatory processes of LN, relapsing LN may involve different injury pathways than de novo LN and may require a different approach to treatment.

Additionally, differences between CR and NR may be due to the intrinsic molecular heterogeneity of LN. That is, some LN may be driven more by autoreactive B cells and NF-κB-dependent cytokines, while LN in other patients may be more T cell dependent.^36^ Finally, the timing of the biopsy relative to an individual's point in the LN flare cycle may contribute to differences between CR and NR. Despite performing kidney biopsies on patients with similar clinical findings, it is generally not known when LN begins in any individual patient with SLE. Furthermore, the progression of parenchymal injury and the kidney's response to injury at the molecular level is almost certainly variable between individuals. Thus, patients who had a rapid clinical response to standard-of-care therapy may have been biopsied earlier in their LN flare than patients who did not respond quickly, and at a time when NF-κB and B cells were highly active. NRs may have evolved to have increased T cell-dependent injury due to a continued inability to remove autoreactive T cells as suggested by suppressed *FADD* and *CD274/PD-L1* expression and increased LFA-1 at biopsy.

It is likely that the duration of LN, its molecular heterogeneity and the timing of biopsy and treatment all contribute to the discordant gene expression patterns in CR and NR. No matter what the explanation, these findings suggest that the pathways active at the time of biopsy may influence response to conventional therapy. Additionally, understanding the active molecular pathways in the kidney when initial treatment is being decided could identify patients who would benefit from novel therapies, such as anti-B cell drugs or drugs that restore immune tolerance.

This study has limitations. The sample size is small with four patients in the NR group and five patients in the CR group. The LN cohort was Hispanic and from Argentina, and the controls were from Ohio. It is possible that some of the molecular heterogeneity of the kidney in LN is influenced by race/ethnicity. Therefore, these results may not be generally applicable to all LN patients. Additionally, whole kidney cortex was studied. Because cortex is mostly tubulointerstitum, these data mainly reflect events occurring in the interstitial compartment. There are likely important differences between the glomeruli and tubulointerstitium. Finally, because transcript expression does not necessarily correlate with protein expression, proteomic evaluation of clinical kidney biopsies could compliment transcript analyses, especially when trying to identify new therapeutic targets.

Nonetheless, our data are consistent with and extend previous studies demonstrating the molecular heterogeneity of LN.^36^ ^50–52^ For example, in a study comparing gene expression profiles of murine and human LN, complement, dendritic cell activation, CTLA4 signalling and antigen presentation pathways were differentially expressed at flare,^50^ similar to the pathways we found to be differentially expressed compared with controls. Additionally, another investigation using microarray analysis of murine kidneys at different disease stages showed a significant increase in inflammatory gene expression at the onset of proteinuria that improved with treatment and returned to baseline levels at clinical remission.^51^ In a recent repeat biopsy study of patients with active LN, serum markers of inflammation increased at flare and decreased after treatment when remission was achieved. However, a poor histological response correlated with higher IL-17 levels at flare and persistently elevated IL-23 levels after treatment, suggesting that the IL-17/IL-23 axis may be an important marker of response.^53^

In summary, these data support the use of molecular pathology to analyse LN biopsies at the time of flare diagnosis. Such analyses could identify patients less likely to respond to standard-of-care therapy, who may do better with a novel drug, and what the novel drug should target. The addition of a molecular evaluation to routine histology would facilitate the personalisation of LN treatment and would be expected to improve short-term response rates and decrease long-term chronic kidney disease. As shown here, this approach is feasible using routinely collected FFPE biopsies. The differentially activated genes and pathways described here will need to be verified in a larger population of patients of different races and ethnicities.
